# Magnesium-based thermoelectric materials and modules for low-temperature applications (below 300°C)

**DOI:** 10.1557/s43577-025-00939-2

**Published:** 2025-06-30

**Authors:** Ran He, Pingjun Ying, Shuo Chen, Zhifeng Ren, Kornelius Nielsch

**Affiliations:** 1 Institute for Metallic Materials, Leibniz Institute for Solid State and Materials Research, Dresden, Germany; 2 Department of Physics and Texas Center for Superconductivity, University of Houston, Houston, USA

**Keywords:** Mg, Thermoelectric, Material availability, Devices, Sustainability

## Abstract

Thermoelectric technology has emerged as a promising solution for direct heat-to-electricity conversion and solid‐state cooling, offering great energy efficiency and environmental impact advantages. However, conventional systems predominantly rely on tellurium-based materials, which are limited by scarcity, high cost, and environmental concerns. This article focuses on tellurium-free thermoelectric modules, with an emphasis on magnesium-based alternatives, including *p*-type MgAgSb and *n*-type Mg_3_(Sb, Bi)_2_, which demonstrate competitive performance at operating temperatures below 300℃. By exploring recent advances in material synthesis, module fabrication, and interface engineering, we highlight the potential of these sustainable materials to achieve high thermoelectric figures of merit while reducing environmental impact. Additionally, the article assesses the performance metrics and durability of these modules and discusses emerging applications in energy harvesting, medical devices, consumer electronics, and more. Finally, we outline future research directions aimed at overcoming remaining challenges, including long-term stability and scalable manufacturing, to pave the way for the widespread adoption of tellurium-free thermoelectric technology.

**Graphical abstract:**

**Figure:**
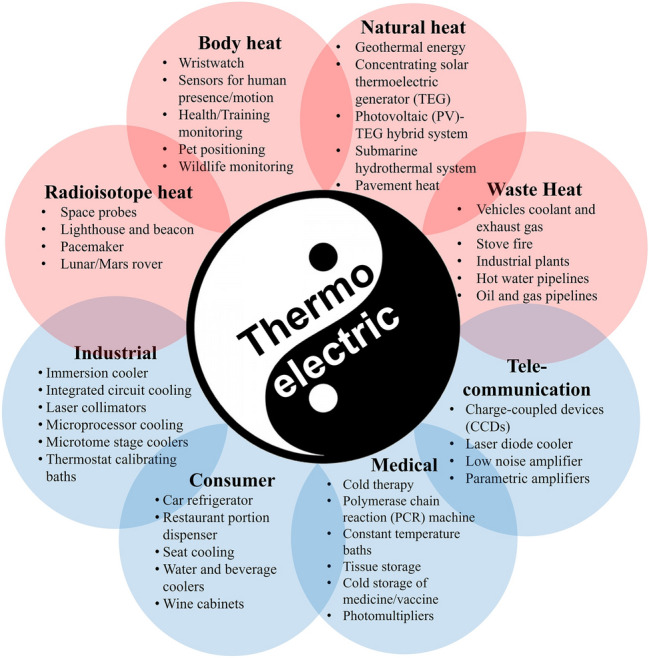
Potential application scenarios of Mg-based Te-free thermoelectric technology

## Introduction

Thermoelectric (TE) technology provides a distinctive approach to energy conversion by enabling the direct transformation of heat into electricity and vice versa. This capability presents promising solutions for both power generation and thermal management.^1^ As global energy demands rise and environmental concerns intensify, utilizing waste heat from industrial operations, automotive exhaust, and even human body heat offers a valuable opportunity to enhance energy efficiency and reduce dependence on conventional energy sources. In addition, solid-state cooling offers versatile cooling options, including noise-free operation, lightweight design, and suitability for small-scale and customized cooling solutions.^2^

Thermoelectric modules capitalize on the Seebeck effect to convert temperature differences directly into electrical voltage. In reverse, applying an electrical current induces cooling via the Peltier effect, enabling these devices to serve a role in thermal management. This dual functionality makes them particularly attractive for applications such as distributed energy solutions for the Internet of Things (IoT) and precise temperature control in medical devices. Central to their performance is the dimensionless figure of merit, *zT*, defined as *zT* = (*S*^2^σ/κ)*T*, where *S* is the Seebeck coefficient, σ the electrical conductivity, κ the thermal conductivity, and *T* is the absolute temperature. The term *S*^2^σ is the power factor.

Although thermoelectric devices offer significant benefits, their broad implementation has been hindered by the limited supply of tellurium (Te)—a scarce, costly element that also raises environmental concerns. The reliance on Te-based materials, such as *p*-type (Bi, Sb)_2_Te_3_ and *n*-type Bi_2_(Te, Se)_3_, restricts large-scale adoption due to Te’s extremely low natural abundance (<0.001 ppm in Earth’s crust) and escalating costs.^3^

To address these challenges, research has increasingly focused on Te-free alternatives. Among the most promising are Mg-based compounds such as *n*-type Mg_3_(Sb, Bi)_2_ and *p*-type MgAgSb.^4^ These materials not only incorporate more abundant elements, but also exhibit favorable thermoelectric properties at low operating temperatures. Their advantages—cost-effectiveness, reduced environmental impact, and competitive performance—make them especially well-suited for applications such as low-grade heat energy harvesting. By focusing on sustainable alternatives, researchers and industries can drive the advancement of thermoelectric technology toward practical and economically viable implementations.

By highlighting recent progress in the field, this article focuses on the advancement and implementation of Te-free thermoelectric materials and modules specifically designed for applications below 300℃, showcasing their potential to revolutionize sustainable energy-conversion solutions. This article is structured to provide a comprehensive analysis of the current state of Te-free thermoelectric modules. We start by exploring the fundamental properties and synthesis processes of *p*-type MgAgSb and *n*-type Mg_3_(Sb, Bi)_2_. Key aspects such as crystal structure, defect chemistry, and transport mechanisms are examined, with emphasis on how factors such as reduced thermal conductivity and optimized charge-carrier transport contribute to enhanced efficiency. Additionally, challenges associated with these materials are discussed, along with strategies for overcoming them.

Then, we shift the discussion to the practical aspects of device fabrication. Here, we review the methods employed in constructing Mg-based modules, including advanced processing techniques such as mechanical alloying and field-assisted sintering (FAST). A particular focus is placed on contact engineering, which plays a crucial role in ensuring reliable interfaces between thermoelectric legs and electrodes. Innovations in soldering, protective coatings, and diffusion barrier technologies are also discussed, highlighting approaches to enhance long-term stability and operational performance.

Afterward, we briefly discuss the diverse applications of next-generation Te-free thermoelectric modules, from energy harvesting and electronic cooling to medical devices and wearable electronics, while also highlighting emerging uses such as cryogenic cooling and hybrid renewable energy systems. It discusses future challenges and advancements, including improving material performance, enhancing fabrication techniques, and leveraging machine learning and additive manufacturing for optimized development.

By offering an accessible overview of Te-free thermoelectric technology, this article aims to serve as a valuable resource for researchers, engineers, and policymakers interested in sustainable energy solutions. The transition away from traditional Te-based systems toward more abundant and environmentally benign alternatives represents a crucial step in the pursuit of efficient, scalable, and sustainable thermoelectric devices.

## Mg-based Te-free materials

Te-free thermoelectric materials—especially those based on Mg—are emerging as promising, sustainable alternatives for converting heat into electricity at lower operating temperatures. Owing to the limited availability and high cost of Te, Mg-based compounds provide a more cost-effective and environmentally friendly solution without sacrificing performance. Notable examples include *p*-type MgAgSb (α-phase) and *n*-type Mg_3_(Sb, Bi)_2_, both exhibiting strong thermoelectric potential for applications below 300℃. This section summarizes their composition, synthesis methods, and thermoelectric properties, as well as the opportunities and challenges associated with their implementation.

## *P*-type α-MgAgSb

**Figure 1:**
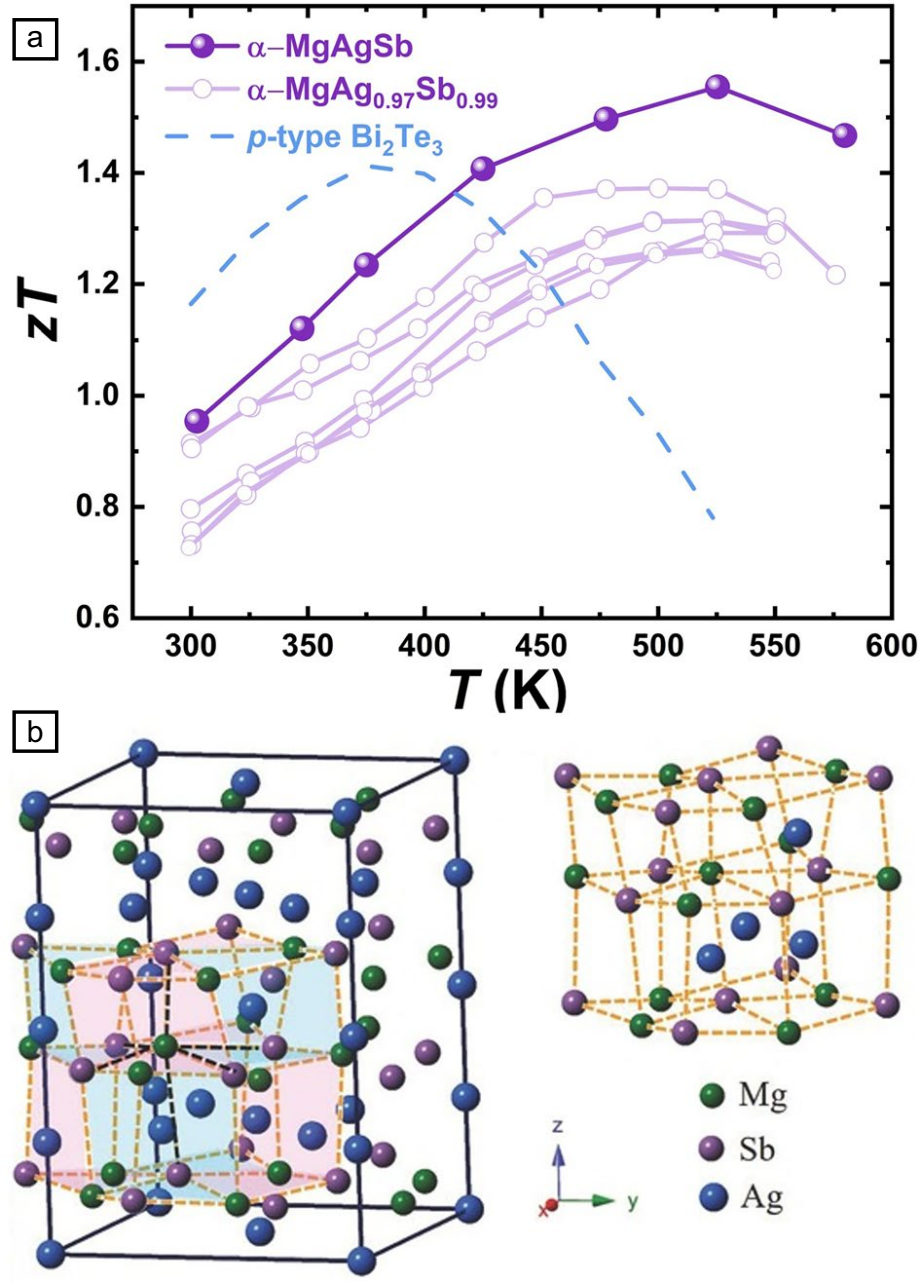
(a) Comparison of *zT* among *p*-type MgAgSb and Bi_2_Te_3_.^9–15^ (b) Crystal structure of α-MgAgSb, looking along the [100] directions. Reprinted with permission from Reference 7. © 2016 Wiley.

The quest for materials that efficiently convert low-grade heat into electricity has positioned MgAgSb as a prime candidate for sub-300℃ applications. Discovered in 2012, this compound leverages abundant elements combined with a complex structural framework, tailored defect chemistry, and favorable electronic properties.^5^ Its exceptional performance stems from its unique ability to simultaneously inhibit heat transfer, similar to glass while promoting electrical conduction similar to a metal—a concept often described as “phonon glass, electron crystal.”^6–8^ Consequently, α-MgAgSb achieves peak thermoelectric figures of merit (*zT*) ranging from 1.0 to 1.6 near 550 K depending on the composition, dopant species, and the processing procedures,^9–16^ as representatively shown in** Figure **1a.

At the atomic scale, α-MgAgSb crystallizes in a complex tetragonal structure (space group I-4c2), where Mg and Sb atoms form a distorted network and Ag atoms occupy interstitial positions (Figure 1b).^5,7^ This arrangement creates a large 24-atom unit cell that effectively scatters phonons, thereby reducing thermal conductivity. However, the structure is temperature-sensitive: it transforms to a β-phase above 588 K and a cubic γ-phase near 663 K.^17^ These phase transitions complicated the synthesis process. Early methods, such as vacuum melting, often yielded samples contaminated with extraneous Sb or Ag_3_Sb.^5^ Recent advances—such as extended heat treatments and a two-step grinding process—enable the production of pure α-MgAgSb, fully realizing its thermoelectric potential.

The material’s high thermoelectric property is enabled by its intrinsically low lattice thermal conductivity (κ_L_). Several factors contribute to this property:

- The weak bonding between Ag and Sb yields a synergistic effect of **lattice softening** and partially **liquid-like motion** of Ag atoms.^18,19^
- The **formation of defects** (e.g., Ag vacancies) disrupts the lattice and further impedes heat flow.^17,20^
- The large atomic unit gives rise to **complex vibrational modes**. The broad spectrum of vibrational modes interferes with one another, limiting thermal energy propagation.^7^

Despite its low κ_L_, α-MgAgSb maintains high electrical conductivity through an electronic structure featuring multiple energy valleys, which enhance electron conduction while preserving high mobility due to minimal electron–phonon interactions. A narrow energy gap (approximately 0.1–0.3 eV) supports this metallic-like behavior.^14^ Additionally, dopants such as Ni, Cu, Li, Na, or Yb are introduced to fine-tune electron concentration.^8,10,12,13^ Further performance enhancements are achieved through nanoscale microstructural engineering. For example, introducing nanopores in MgAgSb could effectively reduce heat propagation.^21^ Defects at different length scales (grain boundaries, dislocations, point defects, etc.) yield synergistic phonon scattering across a broad range of frequencies, all while minimally affecting electron transport. Advanced imaging has revealed localized strain fields around the defects that further inhibit thermal conduction.^10,21^

Beyond its excellent thermoelectric performance, MgAgSb exhibits remarkable mechanical durability.^22^ Its nanostructured design significantly enhances resistance to crack propagation, rendering it far more robust than many conventional brittle thermoelectric materials. However, challenges persist—particularly in managing phase transitions during synthesis. In summary, MgAgSb marks a significant advancement in sustainable energy technology, offering a promising strategy for converting waste heat into electricity despite persistent challenges related to phase stability.

## *N*-type Mg_3_(Sb, Bi)_2_

Another leading candidate for low-temperature thermoelectric applications is the *n*-type Mg_3_(Sb, Bi)_2_-based Zintl phase compound. These materials combine cost-effectiveness, environmental sustainability, and tunable electronic properties, making them attractive alternatives to conventional Bi_2_Te_3_-based systems. Mg_3_(Sb, Bi)_2_ exploits its unique crystal structure and defect chemistry to achieve competitive performance.

**Figure 2:**
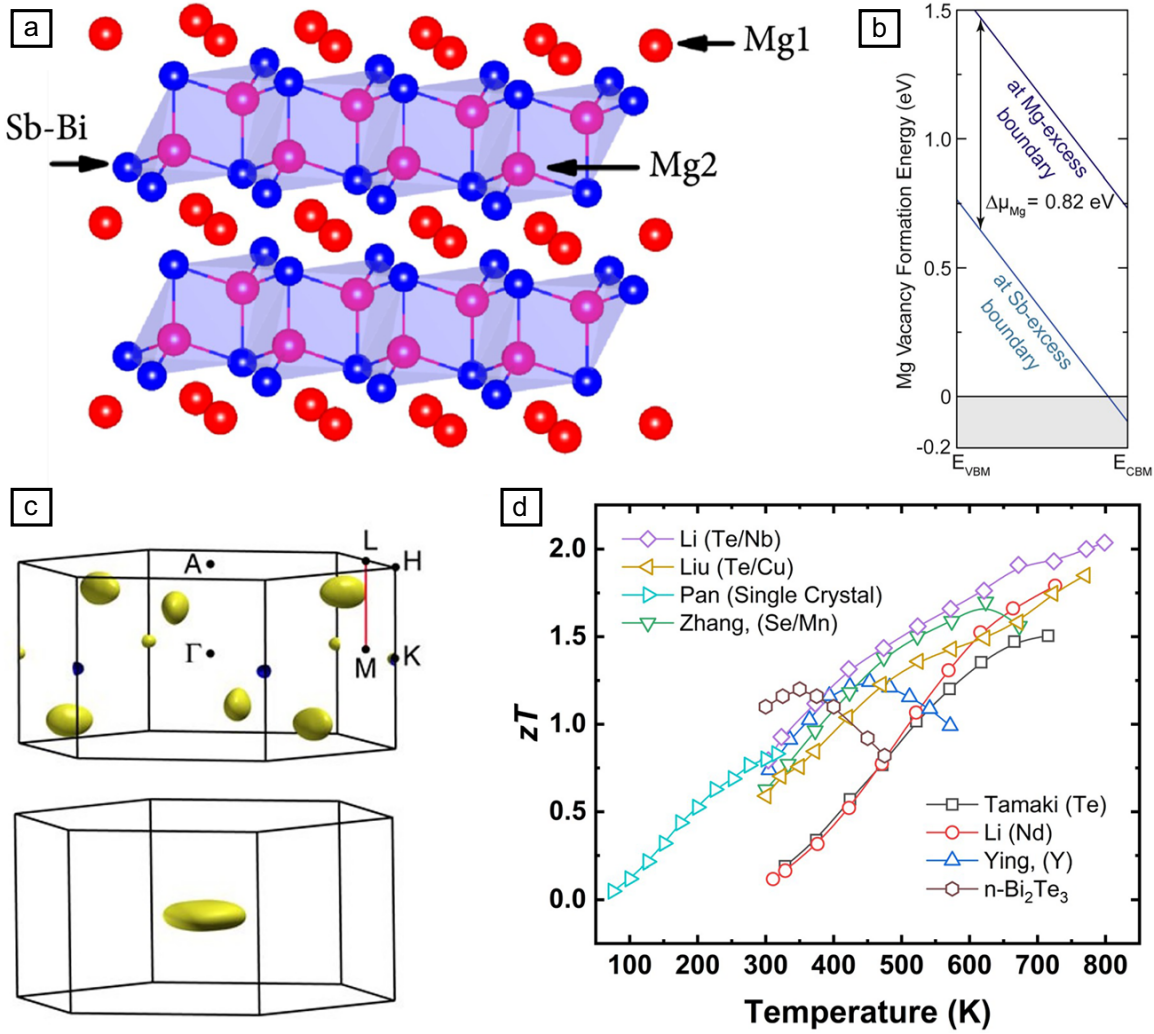
(a) Lattice structure of Mg_3_(Sb, Bi)_2_. (b) Formation energy of Mg vacancies under Mg-rich or Sb-rich conditions. (c) Fermi surface of (top) *n*-type and (bottom) *p*-type Mg_3_Sb_2_ showing the enhanced carrier pockets in *n*-type materials. (d) Comparison of *zT* among several representative Mg_3_(Sb, Bi)_2_ materials.^26,27,34–38^ (a) Adapted from Reference 31. CC BY 4.0. (b) Reprinted with permission from Reference 24. © 2025 Elsevier. (c) Reprinted from Reference 25. CC BY 4.0. VBM, valence-band maximum; CBM, conduction-and minimum.

For instance, Mg_3_Sb_2_ features a layered structure in which covalent [Mg_2_Sb_2_]^2–^ polyanion layers are aligned along the *c*-axis. This arrangement imparts significant anisotropy in both electronic and thermal transport (**Figure** 2a): strong covalent bonding within the *ab*-plane contrasts with weaker ionic interactions along the *c*-axis, resulting in a lower lattice thermal conductivity (κ_L_). In single crystals, the Hall mobility in the *ab*-plane is 75% higher, and the thermal conductivity is 50% lower than along the *c*-axis, highlighting the importance of structural anisotropy.^23^

The electronic properties of Mg_3_(Sb, Bi)_2_ are largely governed by native defects, particularly Mg vacancies (V_Mg_^2−^), which act as electron acceptors.^24^ In stoichiometric compositions, these vacancies position the Fermi level near the valence band, resulting in *p*-type behavior. However, by synthesizing under Mg-rich conditions, vacancy formation is suppressed, shifting the Fermi level toward the conduction band and promoting *n*-type conduction (Figure 2b). Such defect engineering is crucial for optimizing carrier concentrations, which in high-performance *n*-type samples generally range from 10^19^ to 10^20^ cm^−3^.

Band structure engineering further enhances performance. As an indirect semiconductor with an approximate bandgap of ~0.6 eV, Mg_3_Sb_2_ exhibits a conduction band with high valley degeneracy (*N*_v_ = 6) near the L–M points, while its valence band has a lower degeneracy (*N*_v_ = 1) at the Γ point (Figure 2c).^25^ This asymmetry benefits *n*-type performance by enabling multiple carrier pockets that enhance electrical conductivity while maintaining a robust Seebeck coefficient.^26^ Partial substitution of Sb with Bi in Mg_3_Sb_2–x_Bi_x_ offers additional benefits: Bi alloying reduces the bandgap and lowers the effective mass (m*), thereby increasing carrier mobility.^27–31^ In addition, theoretical (first-principles simulations) and experimental (neutron/x-ray scattering) revealed the ultralow κ_L_ of Mg_3_Sb_2_ and Mg_3_Bi_2_ arises from undersized Mg cations weakening interlayer Mg–X bonds, which softens and flattens transverse acoustic phonons, dramatically enhancing anharmonic three-phonon scattering.^32,33^ Concurrently, the resulting mass and strain fluctuations enhance phonon scattering, reducing κ_L_ by roughly 50% at optimal Bi concentrations ((*x* between 0.5 and 1.5). This compositional tuning can yield a peak *zT* of about 2 at high temperatures and around 0.8 near room temperature, positioning Mg_3_(Sb, Bi)_2_ as a competitive alternative to Bi_2_Te_3_ (Figure 2d).^26,27,34–38^

*N*-type Mg_3_Sb_2_ thermoelectrics have benefited from a tiered approach to aliovalent engineering, in which successive dopant families build on one another to optimize carrier concentration, mobility, and phonon scattering. The first development came from chalcogen substitution and band‐engineering via bismuth alloying. Introducing Te or Se at Sb sites creates shallow donor levels that raise electron concentration, while partial replacement of Sb by Bi narrows the bandgap, and promotes band convergence and phonon scattering.^28,39^ These Mg_3_Sb_2–x_Bi_x_ solid solutions deliver peak *zT* ~ 1.5 near 700 K.^26^ Subsequently, transition‐metal codoping and grain‐boundary modifiers further enhance mobility and electrical conductivity. For example, adding Mn simultaneously increases carrier density and the Hall mobility, pushing *zT* to 1.7 at 623 K in Mg_3.15_Mn_0.05_Sb_1.5_Bi_0.49_Se_0.01_.^36^ Likewise, mixtures with Nb, Mo, Cu, or Co, or even metallic compounds such as Mg_2_Cu, refine grain boundaries to reduce interfacial resistance and further improve transport properties.^30,34,40–42^ Rare earth dopants leverage their close ionic radii to Mg^2^⁺ to maximize mobility at high carrier densities. Y-doped Mg_3_Sb_0.6_Bi_1.4_, for example, has achieved an average *zT* of approximately 1.1 from room temperature up to 573 K,^34^ while Nd‐substituted variants approach 1.8 at ~725 K—all with minimal mobility penalties from lattice distortion.^35^ Finally, metallic nano‐inclusions provide a synergistic boost by simultaneously scattering mid‐to‐long‐wavelength phonons and supporting high electrical conductivity. The inclusion of Nb or Ta nanoparticles in Mg_3_Sb_1.5_Bi_0.49_Te_0.01_ yields average *zT * > 1.5 across the mid‐temperature range and achieves a record peak *zT* = 2.04 at 798 K.^37^

The synthesis method plays a critical role in determining both microstructure and performance. Techniques such as ball milling followed by spark plasma sintering (SPS) yield dense pellets with well-controlled stoichiometry, while methods such as Bridgman or flux growth produce single crystals with minimal grain-boundary scattering.^27,43,44^ Microstructural engineering approaches, including the introduction of dislocation networks via mechanical deformation, further enhance phonon scattering without adversely affecting carrier mobility. Moreover, the formation of secondary phases, such as MgO at grain boundaries during sintering, contributes to additional reductions in κ_L_ through interface scattering.

Thermal and environmental stability are significant challenges for Mg_3_(Sb, Bi)_2_. Exposure to moisture and oxygen can lead to the formation of Mg(OH)_2_ and elemental Sb/Bi, which degrade its thermoelectric properties.^45^ Strategies to mitigate these effects include the application of hydrophobic coatings such as boron nitride,^46^ and the use of atomic layer deposition (ALD) to apply protective oxide layers—both of which significantly enhance stability during thermal cycling.^47^ Additionally, the mixture with materials such as Mn or MgB_2_ increases the formation energy of Mg vacancies, reducing Mg loss during operation.^48,49^

Looking ahead, further enhancements in environmental stability, scalable synthesis, and novel compositional exploration are essential. Promising directions include developing high-temperature-resistant hydrophobic coatings and adopting cost-effective production methods, such as self-propagating synthesis, to facilitate large-scale manufacturing. Investigating composite systems—for instance, integrating Mg_3_(Sb, Bi)_2_ with carbon nanotubes—could also yield significant performance improvements.^50^ Recently, single-crystalline Mg_3_Bi_2_ exhibits extraordinary ductility, sustaining up to 100% tensile strain along the (0001) plane, due to low-energy slip systems and dynamic Mg–Bi bonding. This unique mechanical flexibility, combined with a high thermoelectric power factor and a *zT* of ~0.65 at room temperature, makes Mg_3_Bi_2_ a promising candidate for flexible thermoelectric devices in wearable and conformal energy-harvesting applications.^51^ With continued R&D *n*-type Mg_3_(Sb, Bi)_2_ is poised to become a cornerstone of sustainable thermoelectric technology, enabling efficient recovery of low-grade waste heat and providing effective solid-state cooling solutions that bridge the gap between laboratory innovation and practical applications.

## Thermoelectric module performance based on MgAgSb and Mg_3_(Sb, Bi)_2_

Recent advancements in Mg-based thermoelectric materials have positioned these systems as leading candidates for sustainable energy harvesting and cooling applications. Their combination of high thermoelectric efficiency, cost-effectiveness, and reduced environmental impact offers a compelling alternative to conventional Bi_2_Te_3_-based modules. This section provides an analysis of device fabrication methodologies, performance benchmarks, contact engineering strategies, long-term stability challenges, and environmental and economic considerations critical to advancing Mg-based thermoelectric technology.

### Module fabrication methods

**Figure 3:**
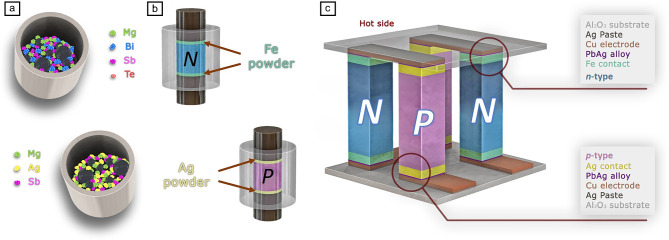
A typical routine for the fabrication of Te-free thermoelectric materials and modules. Adapted from Reference 4. CC BY 4.0.

**Figure** 3a shows a typical process of fabricating high-quality MgAgSb and Mg_3_(Sb, Bi)_2_ modules.^4^ Ball-milled powders are consolidated into dense pellets using a field-assisted sintering technology (FAST) at respective conditions (500–800℃ under 50–80 MPa for Mg_3_(Sb, Bi)_2_; ~300℃ under 80–120 MPa for MgAgSb). During consolidation, the thermoelectric materials are co-sintered with their respective contact layers in a unified stage to establish strong interfacial bonding. For *n*-type components, contact materials are employed such as Fe, stainless steel, and Ni,^52–55^ while *p*-type legs use Ag or MgCuSb.^56,57^ Finally, Sn-based solders connect the thermoelectric legs to Cu electrodes.

### Contact engineering

Contact engineering is pivotal in enhancing both the performance and long-term durability of thermoelectric devices by ensuring robust, low-resistance interfaces between thermoelectric conversion materials and electrodes. Recent advances have focused on systematically addressing key challenges such as high contact resistivity, weak bonding strength, and thermal instability. For instance, Wu et al. implemented a “like-bonds-like” strategy by identifying Fe as an ideal matrix element and then alloying it with small amounts of Mg, Cr, and Ti.^52^ This approach produced ternary alloys (Fe_7_Mg_2_Cr and Fe_7_Mg_2_Ti) that not only achieve shear strengths exceeding 40 MPa, but also maintain specific contact resistivities below 5 μΩ cm^2^ when interfaced with Mg_3_(Sb, Bi)_2_. Such engineered contacts provide remarkable mechanical and electrical stability under high-temperature conditions. However, interfacial degradation remains a critical bottleneck in other systems. For example, in *p*-type MgAgSb devices, Sb migration toward Ag contacts under thermal gradients leads to the formation of an intermediate Ag_3_Sb layer, which degrades the bonding strength due to the mismatch of the coefficient of thermal expansion.^47^ To overcome this, alternative contact materials like MgCuSb have been developed, offering enhanced oxidation resistance and improved mechanical adhesion.^57^

### Performance metrics and comparison

**Figure 4:**
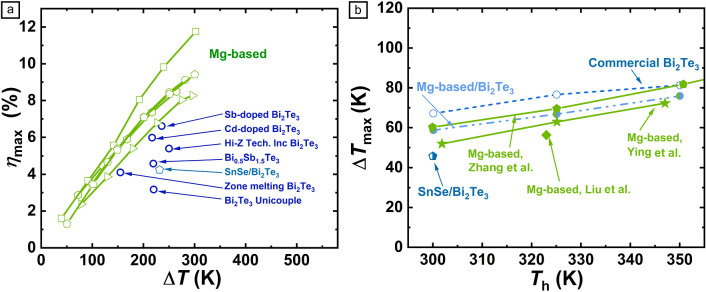
Comparison of module performance for (a) power generation and (b) cooling. Adapted from Reference 34. CC BY 3.0. Reprinted with permission from Reference 15. © 2025 Elsevier.

Mg-based modules exhibit exceptional performance across power generation and cooling applications (**Figure** 4). A 7-pair module combining *p*-type MgAgSb (*zT*_avg_ = 1.4, 300–573 K) and *n*-type Mg_3.285_Nb_0.015_SbBi_0.9975_Te_0.0035_ (*zT*_avg_ = 1.4) achieved a record 12% conversion efficiency at a temperature gradient of Δ*T* = 287 K (*T*_hot_ = 583 K, *T*_cold_ = 296 K).^15^ This efficiency not only surpasses Bi_2_Te_3_ modules under similar conditions, but also ranks among the highest reported for single-stage thermoelectric generators across a broad temperature range (300–950 K). Under natural convection cooling, Mg-based modules generate 400% higher output power than Bi_2_Te_3_ due to their intrinsically low thermal conductivity (<2 W/m·K), which minimizes parasitic heat losses. In cooling applications, MgAgSb/Mg_3_(Sb, Bi)_2_ modules achieve a maximum temperature difference (Δ*T*) of 200 K at a hot-side temperature of 390 K. This capability is particularly advantageous for thermal management in high-heat-flux electronics. Additionally, the coefficient of performance (COP) for cooling reaches 4.3 at *T*_cold_ = 400 K, outperforming Bi_2_Te_3_ (COP = 2.76) and highlighting their suitability for energy-efficient refrigeration.^34^

Output power per module is another critical metric, particularly when heat is abundant or wasted. At the materials level, power output is enhanced by improving the power factor. However, for Mg-based materials, the intrinsic materials’ properties (such as band structure) limit further enhancements in the power factor. Alternatively, power output can be increased at the module level by either boosting the output voltage or reducing the internal resistance. This can be achieved by incorporating more leg pairs to raise voltage or by designing legs with shorter heights and larger cross-sectional areas to lower resistance. The optimal strategy depends on specific application requirements and fabrication constraints. Currently, most Mg-based modules focus on efficiency by employing taller legs. While this approach improves efficiency, it also increases internal resistance, thereby reducing the overall output power. Future research should, therefore, target the development of module designs that achieve a better balance between efficiency and power output.

### Long-term stability and environmental considerations

**Figure 5:**
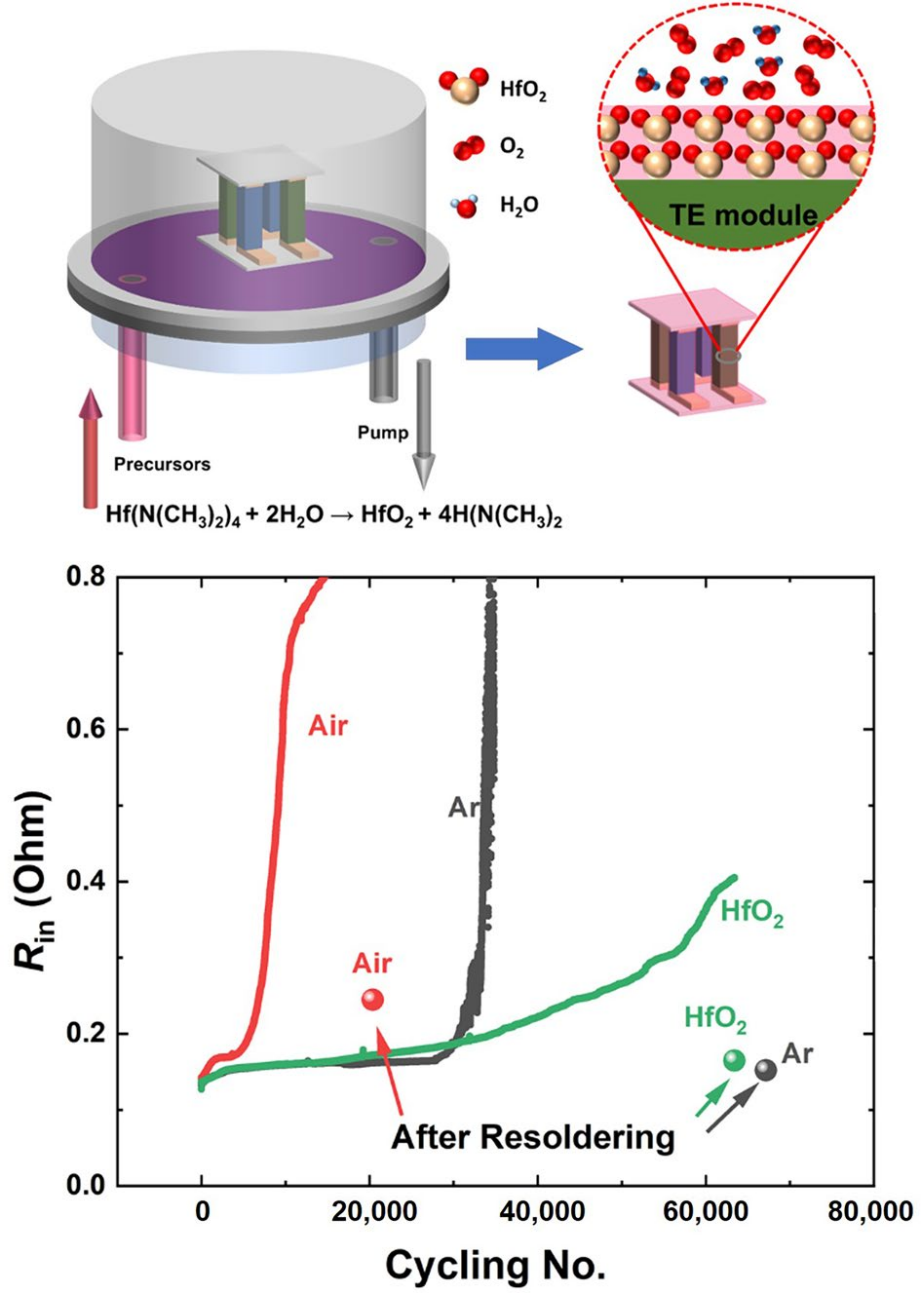
A schematic of the atomic layer deposition principle (top) and its protective power for thermoelectric (TE) modules (bottom). Adapted from Reference 47. CC BY 4.0.

Mg-based modules face intrinsic stability challenges, particularly Mg loss through evaporation at elevated temperatures and phase separation along grain boundaries for *n*-type Mg_3_(Sb, Bi)_2_. To counter these issues, targeted doping strategies have been developed. For example, in comparison to the Te- or Se-doped compounds, the ones that are doped by rare earth elements display better stability such as Y, Yb, or Er.^29,58,59^ To further improve robustness during air operation, ALD coatings of either Al_2_O_3_ or HfO_2_ (300-nm thick) are applied to protect the modules from environmental degradation.^47^ Notably, HfO_2_ demonstrates superior stability than Al_2_O_3_ upon thermal cycling in air, attributed to its enhanced thermodynamic resilience. Acting as effective diffusion barriers, these coatings significantly reduce performance degradation; HfO_2_-coated modules retained 93% of their initial output power after 65,000 thermal cycles in air, compared to a 64% loss in uncoated modules after only 20,000 cycles, as shown in** Figure** 5. Moreover, advanced brazing techniques are critical, as resoldering degraded legs restored 95% of performance in ALD-protected modules.^47^

### Future directions and challenges

Although Mg-based thermoelectric modules show significant promise, several challenges remain. On the materials side, enhancing performance is still difficult. One promising strategy is to reduce oxygen content, such as by pre-reducing powders before sintering, to improve material quality. Another compelling direction is to explore thermoelectric properties below room temperature, to identify materials suitable for cryogenic applications. At the device levels, one major concern is the development of alternative contact materials beyond conventional options like Ag and Fe, aiming to reduce both costs and degradation. While numerous innovative contact materials have been proposed, they still require rigorous, long-term stability testing. For instance, a recent study suggests that the MgAgSb/Sb contact exhibits an unexpected reduction in contact resistance after prolonged air exposure, highlighting a promising avenue for future research.^60^ Another critical challenge is scaling ALD coatings for mass production—a topic that has yet to be investigated. Moreover, module-level issues arise in cooling applications, where the interaction of working current with moisture or ice over extended periods remains largely unexplored. Sustainability considerations also demand effective recycling strategies for these modules at the end of their operational life, which is currently unclear for Mg-based modules and warrants further research to develop eco-friendly protocols. In addition, recent findings indicate that Mg-based materials display excellent plasticity, allowing them to be bendable or machinable into various shapes, making them highly promising for applications requiring non-cuboid thermoelectric materials.^51^ This advancement opens up opportunities in fields such as human body heat harvesting. Overall, continued innovation could enable MgAgSb and Mg_3_(Sb, Bi)_2_-based modules to redefine sustainable energy technologies by offering high efficiency, environmental compatibility, and cost-effectiveness for applications ranging from wearable electronics to industrial waste heat recovery.

## Applications and future prospects

**Figure 6:**
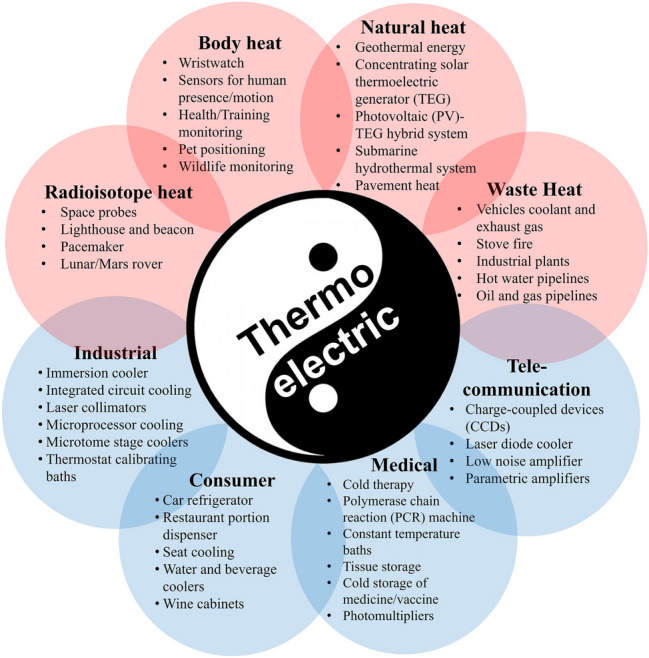
Potential application scenarios of thermoelectric technology. TEG, thermoelectric generator; PV, photovoltaic; PCR, polymerase chain reaction.

Recent advances in Mg-based thermoelectric modules reshape energy solutions by offering sustainable, efficient, and versatile alternatives across various fields (**Figure** 6). These modules are increasingly applied in medical devices, consumer products, wearable electronics, and IoT systems.^61–65^ In the medical arena, they enable precise temperature control for treatments, such as cold therapy, enhance laboratory instruments such as polymerase chain reaction (PCR) machines, and provide stable conditions for sensitive diagnostic tools and imaging systems. Consumer markets also benefit from innovations such as car refrigerators, seat cooling systems, and compact home appliances that operate silently and efficiently. Furthermore, wearable electronics harness body heat to generate electrical energy, powering devices such as smartwatches, pulse oximeters, and health monitors, while IoT sensors utilize ambient temperature differences to reduce dependence on conventional batteries. Beyond these established applications, thermoelectric modules are finding roles in emerging fields such as smart building integration,^66^ cryogenic cooling systems,^67^ space missions through radioisotope thermoelectric generators,^68^ and hybrid renewable power systems such as solar and geothermal power,^69,70^ underscoring their broad potential to transform thermal management and energy harvesting.

Looking forward, the future of thermoelectric technology promises to evolve significantly as researchers address challenges related to both new materials and module fabrication techniques. While conventional materials such as Bi_2_Te_3_ have demonstrated notable performance at room temperature, issues concerning toxicity, limited availability, and stagnant efficiency have driven the search for more sustainable alternatives such as Mg-based compounds. These emerging materials offer enhanced sustainability, reduced costs, and improved overall performance, especially when modern data-driven approaches, such as machine learning integrated with extensive materials databases, are employed to rapidly optimize their properties.^71,72^ At the same time, the complexity of current module fabrication processes, marked by intricate assembly and rigid structures, presents a barrier to scalability and broader adoption.^73,74^ Innovations in additive manufacturing, particularly 3D printing, offer promising solutions by enabling the production of thermoelectric components with intricate geometries that better conform to diverse surfaces.^75–78^ By simplifying fabrication processes and tailoring these new materials for mass production, production costs could be significantly lowered, ultimately facilitating integration with renewable energy systems and contributing to a more resilient, environmentally friendly energy landscape.

## Conclusions

Te-free thermoelectric modules represent a significant leap forward in sustainable energy technology. By harnessing abundant and environmentally benign materials such as *p*-type MgAgSb and *n*-type Mg_3_(Sb, Bi)_2_, these modules address the critical limitations of conventional Te-based systems while delivering comparable, and in some cases superior, performance for low-temperature applications. The advancements in material design, coupled with innovative fabrication and contact engineering techniques, have resulted in devices that offer high conversion efficiency, robust mechanical stability, and extended operational lifetimes. Despite these achievements, challenges remain—particularly in scaling production, optimizing long-term durability under operational stresses, and integrating these modules into a broader array of applications. Future research, leveraging emerging tools such as machine learning for materials discovery and advanced manufacturing technologies such as additive printing, is poised to drive further improvements. As global energy demands grow and environmental imperatives intensify, the continued development and refinement of Te-free thermoelectric modules will play an essential role in the evolution of clean, efficient, and sustainable energy-conversion systems.
